# Identification of key vaginal microbial signatures and immune remodeling associated with HR-HPV clearance following Kushen Gel treatment: a longitudinal analysis

**DOI:** 10.3389/fmed.2026.1841519

**Published:** 2026-07-02

**Authors:** Ying Wang, Shuheng Pan, Fengying Zhang, Huimin Ma, Rihan Wu, Jing Zhang, Xia Li

**Affiliations:** Hohhot Maternal and Child Health Care Hospital, Hohhot, Inner Mongolia, China

**Keywords:** high-risk HPV, IFN-*γ*, Kushen Gel, *Lactobacillus*, localized immunity, vaginal microbiota

## Abstract

**Background:**

Persistent high-risk human papillomavirus (HR-HPV) infection drives cervical carcinogenesis, often exacerbated by vaginal dysbiosis and localized immune dysfunction. Kushen Gel shows clinical promise, yet its impact on microbial-immune crosstalk during HR-HPV clearance remains unclear. This study elucidates the microbial remodeling and immune shifts associated with Kushen Gel-mediated HR-HPV regression.

**Methods:**

A retrospective analysis of 230 vaginal swabs (130 pre-treatment, 100 post-treatment) via 16S rRNA sequencing characterized community structural shifts. Subsequently, a prospective cohort of 35 patients with persistent HR-HPV infection (defined as laboratory-confirmed positive HR-HPV DNA for ≥12 months) validated clinical outcomes (HR-HPV clearance, vaginal pH, Nugent scores) alongside paired 16S rRNA sequencing and ELISA-based quantification of cervicovaginal cytokines (IL-8, IL-6, TNF-*α*, IFN-*γ*).

**Results:**

Kushen Gel intervention significantly decreased microbial alpha diversity and was associated with a distinct beta-diversity shift toward a stable, *Lactobacillus*-dominant state. Models (LEfSe, Random Forest) identified a marked reduction in pathobionts (*Gardnerella*, *Sneathia*, *Prevotella*) post-treatment. In the prospective cohort, the HR-HPV clearance rate reached 82.9% (29/35) after three menstrual cycles, synchronized with significant reductions in mean vaginal pH (4.85 ± 0.42 to 4.12 ± 0.35, *p* < 0.001) and an 85.7% Nugent score normalization rate. Crucially, Kushen Gel treatment was associated with a profound shift from a pro-inflammatory to an anti-viral immune microenvironment. Pro-inflammatory markers (IL-8, IL-6, TNF-*α*) plummeted significantly (*p* < 0.0001), while anti-viral IFN-*γ* exhibited a robust increase (3.2 ± 1.1 to 18.6 ± 5.4 pg./mL, *p* < 0.0001), particularly in responders. *Lactobacillus* abundance positively correlated with IFN-*γ* (*r* = 0.68) and inversely with IL-8 (*r* = −0.54).

**Conclusion:**

Kushen Gel is associated with HR-HPV clearance and concurrent vaginal microenvironment remodeling, marked by suppressed anaerobic-driven inflammation and an enhanced IFN-*γ*-associated anti-viral niche dominated by *Lactobacillus*. These findings biologically support using Kushen Gel to manage vaginal dysbiosis and HR-HPV regression.

## 1 Background

High-risk human papillomavirus (HR-HPV) infection is a primary etiological factor for the development of cervical intraepithelial neoplasia (CIN) and cervical cancer, posing a significant threat to women’s reproductive health worldwide (1, 2). While the host immune system clears most HPV infections spontaneously, persistent HR-HPV infection is closely associated with the disruption of the vaginal microenvironment (3, 4). Emerging evidence suggests that the vaginal microbiota plays a pivotal role in the natural history of HPV infection (5, 6). A healthy vaginal niche, typically dominated by *Lactobacillus* species, provides a biological barrier against pathogens; conversely, decreased *Lactobacillus* abundance and increased microbial diversity—often characterized by the overgrowth of anaerobic bacteria such as *Gardnerella* and *Sneathia*—are correlated with higher rates of HPV acquisition and persistence (7–9).

Crucially, the interplay between vaginal dysbiosis and the host mucosal immune response is a critical determinant of these clinical outcomes. Persistent infection is often characterized by a pro-inflammatory microenvironment, where the overgrowth of anaerobic pathobionts triggers the release of various cytokines, such as interleukin-8 (IL-8), IL-6, and tumor necrosis factor-alpha (TNF-*α*) (10, 11). These inflammatory mediators can compromise the cervical epithelial barrier, thereby facilitating viral entry and persistence. Conversely, a Lactobacillus-dominant niche supports the production of interferon-gamma (IFN-*γ*), a key cytokine that orchestrates local anti-viral immunity and promotes viral clearance (12). Despite the recognized importance of these immune signatures, the potential of therapeutic interventions to achieve HR-HPV regression by concurrently modulating both the microbial architecture and the localized cytokine network remains to be fully elucidated.

To address this gap, Kushen Gel, a botanical formulation derived from the dried roots of *Sophora flavescens Ait.*, has gained widespread clinical utilization due to its multi-targeted pharmacological profile. While primarily recognized for its matrine and oxymatrine content, recent phytochemical characterizations have identified over 200 compounds within the extract, including key quinolizidine alkaloids such as sophocarpine and sophoridine, which work synergistically to exert anti-inflammatory, antimicrobial, and antiviral effects (13–15). Recent high-quality evidence-based studies, including a 2025 systematic review of nine RCTs, have demonstrated that Kushen gel-based interventions significantly improve HR-HPV clearance rates (RR = 1.51) and markedly reduce viral load (MD = −0.70) compared to conventional treatments (16). Mechanistically, original experimental research has revealed that these active alkaloids promote viral regression by downregulating the expression of HPV-related E6 and E7 oncoproteins, which in turn restores the p53 and Rb tumor-suppressor pathways, inducing G2/M arrest and apoptosis in infected epithelial cells (17, 18). Furthermore, previous findings indicate that Kushen Gel functions as an immunomodulator; it activates Toll-like receptor 9 (TLR9) signaling and correlates with an enhanced anti-viral immune response by enhancing the production of cytokines such as IFN-*γ* and IL-12 (19, 20). Clinical network meta-analyses also rank Kushen Gel combined with interferon as a top-tier intervention for clinical effectiveness and long-term virus clearance (21). However, most existing studies focus on macro-level clinical observations or isolated molecular pathways, and the specific longitudinal alterations in the microbial community architecture and the key functional taxa associated with Kushen Gel treatment remain insufficiently characterized.

In our current study, we first conducted a large-scale retrospective analysis using 16S rRNA gene sequencing on 230 vaginal swab samples to decipher the shift in microbial diversity and community composition. To further validate the clinical relevance of these microbial changes, we subsequently performed a prospective study on a cohort of 35 patients to observe the dynamic changes in HR-HPV clearance rates, clinical physiological indicators, and localized cytokine profiles. This study aims to provide a comprehensive understanding of how Kushen Gel promotes a transition from a dysbiotic, pro-inflammatory state to a Lactobacillus-dominant, anti-viral microbiome, thereby supporting the clinical clearance of HR-HPV.

## 2 Materials

### 2.1 Study population and sample collection

The study was conducted in two sequential phases at Hohhot Maternal and Child Health Care Hospital: a large-scale retrospective microbial analysis followed by a prospective clinical validation.

#### 2.1.1 Retrospective cohort for 16S sequencing

A total of 230 vaginal swab samples were retrospectively collected from patients diagnosed with HR-HPV infection at Hohhot Maternal and Child Health Care Hospital between June 1, 2023 and June 1, 2025. The cohort included 130 samples obtained before treatment (before group) and 100 samples obtained after a complete course of Kushen Gel treatment (after group).

#### 2.1.2 Prospective validation cohort

To validate the clinical efficacy and microbial remodeling, a single-arm, prospective longitudinal study was conducted. Sample size was calculated based on an expected clearance rate of 60%, providing 80% power at a 5% significance level, which determined a minimum requirement of 44 participants. Considering a potential 10% dropout rate, 50 patients were initially enrolled.

Inclusion criteria were: (i) age 18–55 years; (ii) persistent HR-HPV infection (defined as laboratory-confirmed positive HR-HPV DNA for a duration of ≥12 months without clearance); and (iii) no use of antibiotics, antifungals, or vaginal medications within the preceding 30 days. Participants received Kushen Gel (5 g per night) for three consecutive menstrual cycles. The Kushen Gel used in this study was commercially obtained from Guiyang Xintian Pharmaceutical Co., Ltd., China. Clinical indicators, including vaginal pH, Nugent score, and HR-HPV status, were recorded at baseline and the 3-month follow-up.

### 2.2 Ethical statement

The study was approved by the Ethics Committee of Hohhot Maternal and Child Health Care Hospital (Approval Number: Hsfybjy2023-18). Written informed consent was obtained from all participants in the prospective cohort.

### 2.3 Library construction and sequencing

#### 2.3.1 PCR amplification

The V3-V4 hypervariable regions of the bacterial 16S rRNA gene were amplified using the universal primers 341F (5’-CCTAYGGG RBGCASCAG-3′) and 806R (5’-GGACTACNNGGGTATCTAAT-3′). The PCR reaction was performed using Phusion® High-Fidelity PCR Master Mix (New England Biolabs, USA). The amplicons were purified using the GeneJET™ Gel Extraction Kit (Thermo Scientific, USA).

#### 2.3.2 DNB library preparation and sequencing

The purified PCR products were used to construct DNA libraries. Briefly, the amplicons were end-paired and ligated with specific adapters. The resulting products were denatured to produce single-stranded DNA, which was then circularized. The single-stranded circular DNA molecules were replicated via rolling circle amplification (RCA) to generate DNA Nanoballs (DNBs). These DNBs were loaded into patterned nanoarrays and sequenced on the MGISEQ-2000 platform (BGI, Shenzhen, China) using the combinatorial Probe-Anchor Synthesis (cPAS) method, generating 250 bp paired-end reads.

### 2.4 Bioinformatic and statistical analysis

Raw sequencing reads were filtered using fastp (v0.19.4) and merged using FLASH (v1.2.11) with a minimum overlap of 10 bp. High-quality sequences were clustered into Operational Taxonomic Units (OTUs) at 97% identity using USEARCH (v7.0). Taxonomic assignment was performed against the SILVA database (v138) using the RDP Classifier with a confidence threshold of 0.8.

Alpha diversity indices (ACE, Chao1, Shannon, Simpson, and Coverage) were calculated using Mothur (v1.30.2). Beta diversity was assessed via Principal Coordinates Analysis (PCoA) and Non-metric Multidimensional Scaling (NMDS) based on Bray-Curtis distances. The significance of community structural differences was tested using Permutational Multivariate Analysis of Variance (PERMANOVA).

Linear Discriminant Analysis Effect Size (LEfSe) was employed to identify significantly enriched taxa between the before and after group. A Random Forest model was constructed to evaluate the diagnostic potential of the identified biomarkers, and the Area Under the Curve (AUC) was calculated using the pROC package in R (v4.4.2). Correlation networks among the genera were analyzed using Spearman’s rank correlation.

### 2.5 Measurement of cervicovaginal cytokines

To evaluate the local immune microenvironment, the concentrations of key cytokines, including interleukin-8 (IL-8), IL-6, tumor necrosis factor-alpha (TNF-*α*), and interferon-gamma (IFN-*γ*), were quantified in vaginal discharge samples. Vaginal swabs collected at baseline and the 3-month follow-up were eluted in 1 mL of sterile phosphate-buffered saline (PBS) and centrifuged at 3,000 rpm for 10 min at 4 °C. The resulting supernatants were harvested and stored at −80 °C until analysis. Cytokine levels were measured using commercial high-sensitivity enzyme-linked immunosorbent assay (ELISA) kits (Invitrogen; Thermo Fisher Scientific, Inc.; Cat# KHC0081, EH2IL6, KHC3011 and KHC4021, respectively) following the manufacturer’s instructions. All samples were assayed in duplicate, and the results were expressed in picograms per milliliter (pg/mL).

### 2.6 Integrative statistical analysis

Clinical data and cytokine concentrations were analyzed using SPSS (v26.0) and GraphPad Prism (v9.0). Continuous variables are presented as mean ± standard deviation (SD). For longitudinal comparisons within the prospective cohort, the paired-sample t-test or Wilcoxon signed-rank test was employed to determine the significance of changes in cytokine levels before and after Kushen Gel treatment. To decipher the relationship between microbial restoration and immune modulation, Spearman’s rank correlation was performed to assess the associations between the relative abundance of dominant bacterial genera and cytokine concentrations. A two-tailed *p* < 0.05 was considered statistically significant.

## 3 Results

### 3.1 Alterations in microbial alpha and beta diversity

A total of 230 vaginal swab samples from HR-HPV infected patients were retrospectively analyzed, comprising 130 samples collected at baseline (before group) and 100 samples collected after Kushen Gel treatment (after group).

**Figure 1:**
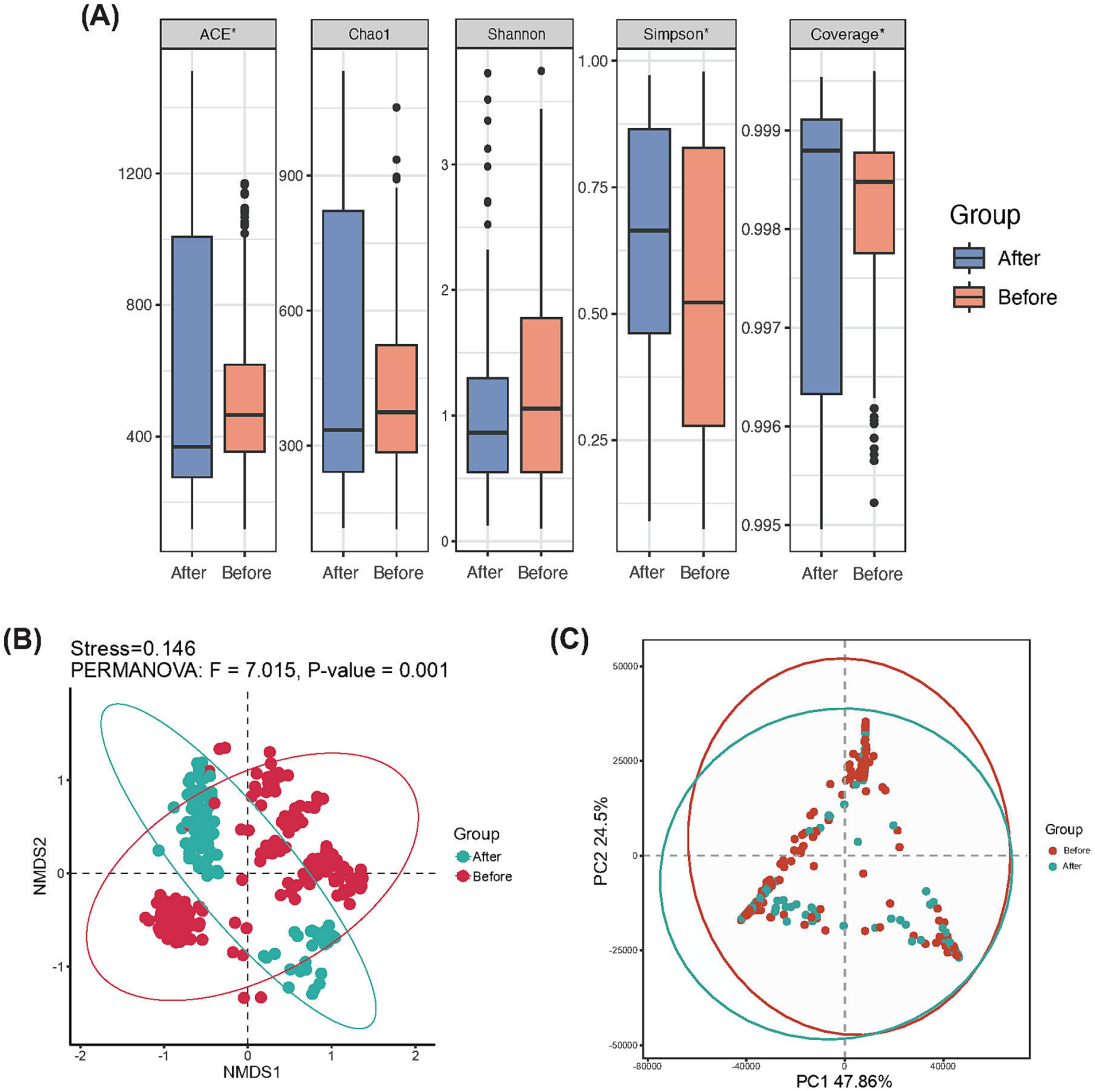
Kushen Gel treatment reshaped the vaginal microbial diversity in patients with HR-HPV infection. **(A)** Boxplots of alpha diversity indices (ACE, Chao1, Shannon, Simpson, and Coverage) in the before and after groups. **(B)** NMDS analysis based on Bray-Curtis distances between the two groups (Stress = 0.146; PERMANOVA: *F* = 7.015, *p* = 0.001). **(C)** Principal coordinates analysis (PCoA) based on Bray–Curtis distances between the two groups.

Alpha diversity analysis demonstrated significant changes in the richness and diversity of the vaginal microbiota following Kushen Gel intervention. The ACE and Chao1 indices, which measure community richness, significantly decreased in the after group compared to the before group. Conversely, the Simpson index showed a significant increase, indicating a more concentrated community structure post-treatment (Figure 1A).

Beta diversity was assessed to visualize the global structural shifts. NMDS analysis revealed a distinct separation between the before and after groups (Stress = 0.146). This separation was statistically significant as confirmed by PERMANOVA (*F* = 7.015, *p* = 0.001) (Figure 1B). Furthermore, PCoA results showed that PC1 and PC2 explained 47.86% and 24.5% of the total variance (Figure 1C), respectively, with a clear migration of samples along the PC1 axis toward a more stable microbial state after treatment.

### 3.2 Taxonomic composition of the vaginal microbiota following Kushen Gel treatment

Based on the 16S rRNA sequencing data from the retrospective cohort, the vaginal microbial composition was characterized across multiple taxonomic levels to identify shifts associated with Kushen Gel intervention.

**Figure 2:**
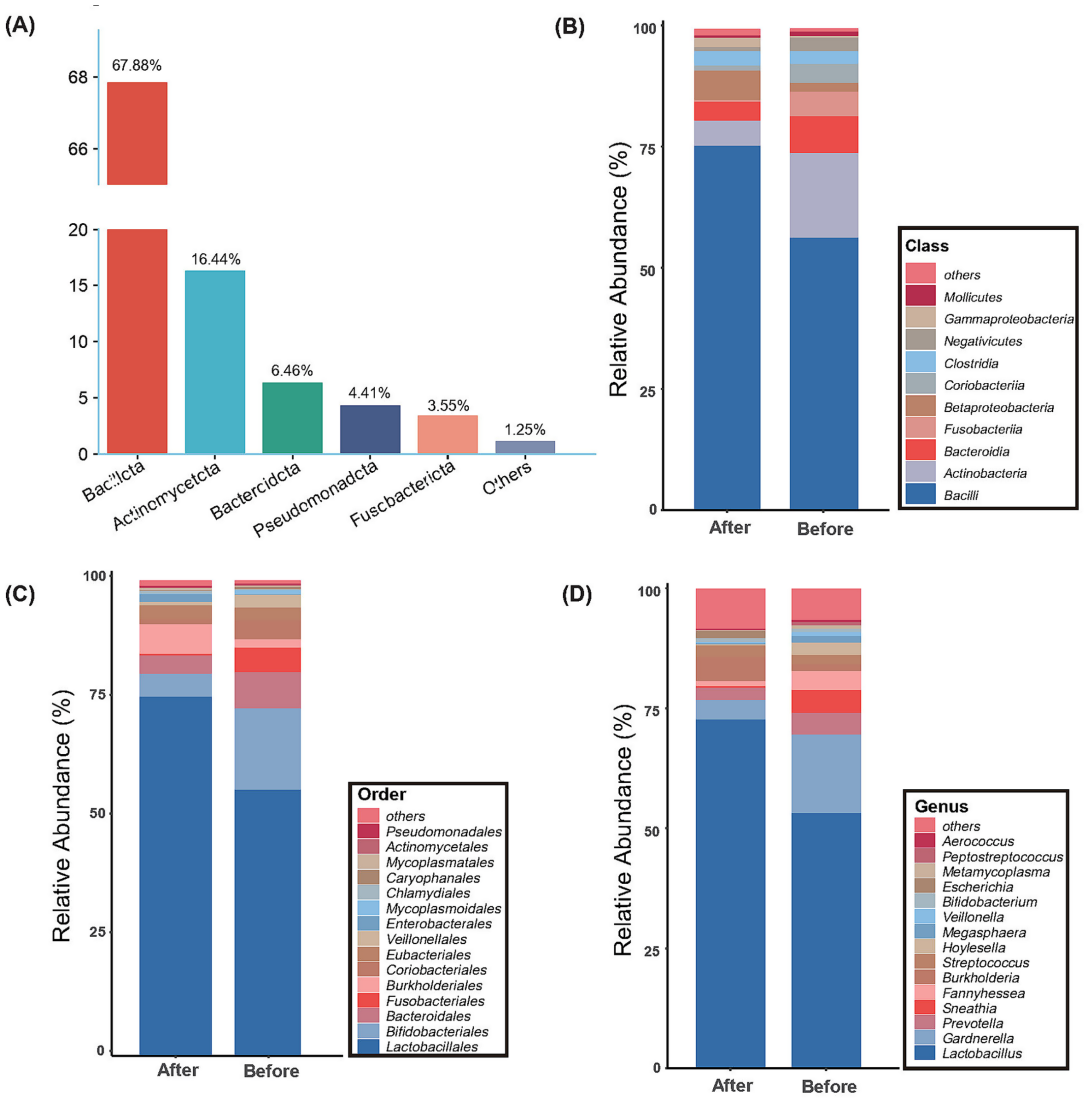
Taxonomic composition of the vaginal microbiota before and after Kushen Gel treatment. **(A)** Relative abundance of dominant bacterial phyla. **(B-D)** Stacked bar plots showing microbial composition at the class **(B)**, order **(C)**, and genus **(D)** levels in the before and after groups.

At the phylum level, *Bacillota* was the most predominant taxon (67.88%), followed by *Actinomycetota* (16.44%) and *Bacteroidota* (6.46%) (Figure 2A). After treatment, the relative abundance of the class *Bacilli* exhibited increase, while taxa typically associated with dysbiosis, such as *Actinobacteria* and *Bacteroidia*, were reduced (Figure 2B). The structural reorganization was further evident at the order level, characterized by a substantial enrichment of *Lactobacillales* and the suppression of anaerobic orders, including *Bacteroidales* and *Fusobacteriales* (Figure 2C). At the genus level, *Lactobacillus* was the primary dominant taxon, and its relative abundance increased following Kushen Gel administration (Figure 2D). Conversely, potential pathobionts, most notably *Gardnerella*, *Prevotella*, *Sneathia*, and *Fannyhessea*, showed a distinct decline in the after group.

These taxonomic shifts suggest that Kushen Gel effectively remodels the vaginal microenvironment by promoting a Lactobacillus-dominant niche while inhibiting anaerobic bacteria linked to HR-HPV persistence.

### 3.3 LEfSe and random forest model analysis

To further identify the specific bacterial taxa that distinguish the vaginal microbiota before and after Kushen Gel treatment, LEfSe and Random Forest analyses were performed.

**Figure 3:**
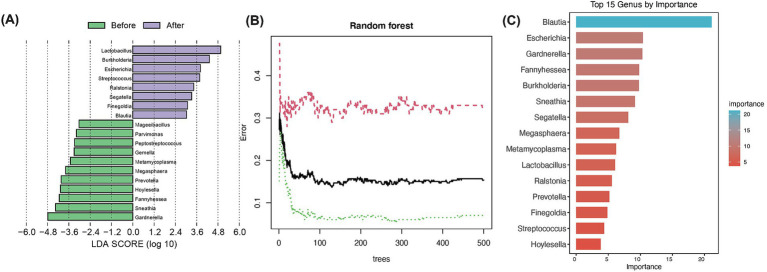
Identification of differential bacterial taxa and biomarker screening using LEfSe and Random Forest analysis. **(A)** Linear discriminant analysis effect size (LEfSe) identifying bacterial taxa that significantly differentiate the before and after groups. **(B)** Random Forest classification model showing the variation of out-of-bag (OOB) error with the number of decision trees. **(C)** Importance ranking of the top 15 bacterial genera identified by the Random Forest model.

LEfSe analysis identified significantly differential taxa between the two groups. Taxa such as *Gardnerella*, *Sneathia*, *Fannyhessea*, and *Prevotella* were highly enriched in the before group. Conversely, *Lactobacillus*, *Burkholderia*, *Escherichia*, and *Streptococcus* showed a significant enrichment in the after group (Figure 3A). The Random Forest model was then constructed to evaluate the classification power of these genera. The model error rate stabilized as the number of decision trees reached approximately 200 (Figures 3B,C). The top 15 genera ranked by their importance in the model included *Blautia*, *Escherichia*, *Gardnerella*, and *Fannyhessea*, highlighting their critical role in defining the community shift.

### 3.4 Predictive performance and abundance comparison

**Figure 4:**
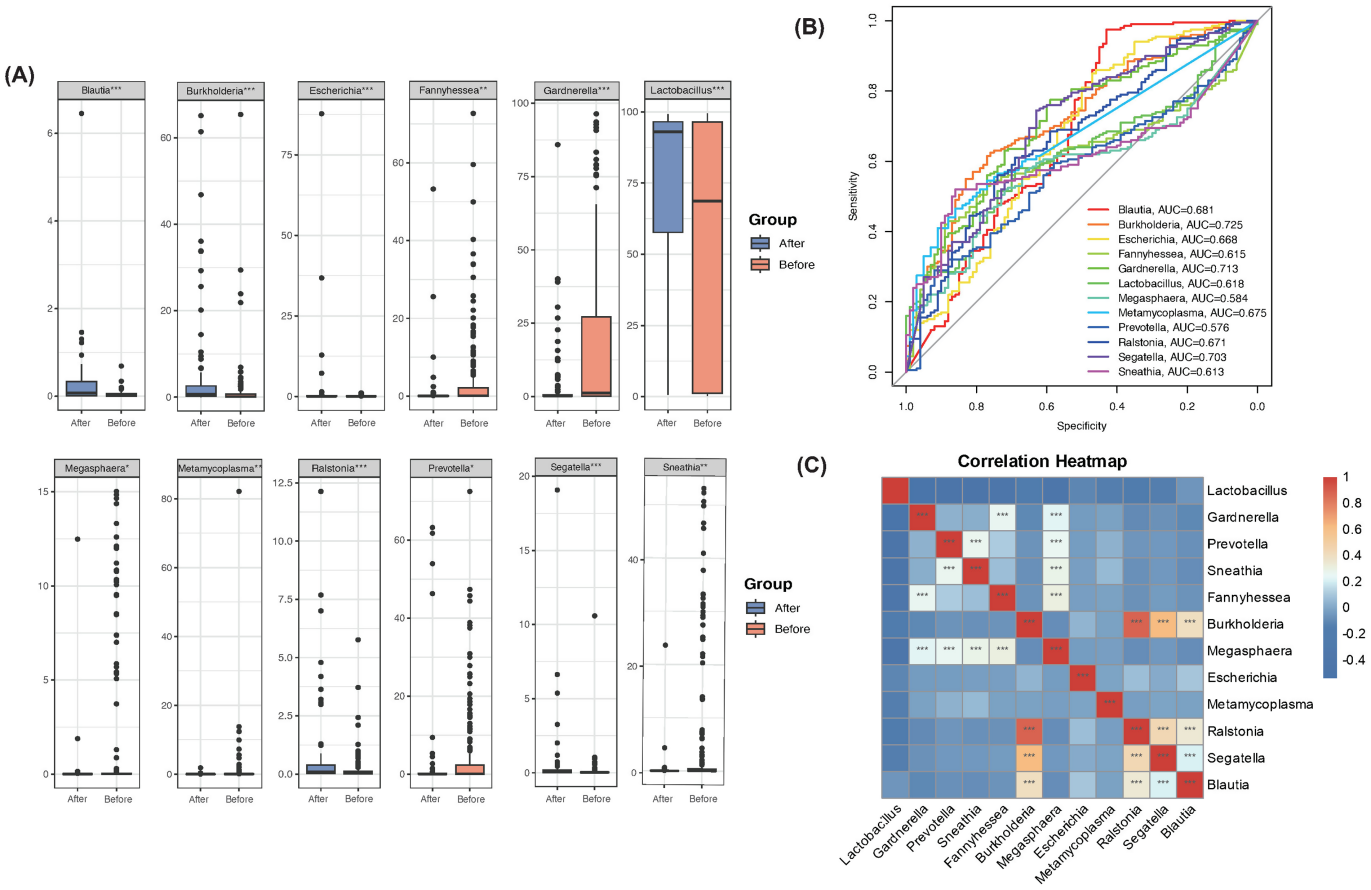
Differential abundance, diagnostic potential, and ecological relationships of key vaginal microbial genera. **(A)** Relative abundance comparison of selected bacterial genera between the before and after groups. *p < 0.05, **p < 0.01, ***p < 0.001. **(B)** Receiver operating characteristic (ROC) curve analysis evaluating the predictive performance of key genera for distinguishing treatment response. **(C)** Spearman correlation heatmap among dominant vaginal microbial genera.

The dynamic changes of the top-ranked genera were confirmed through relative abundance comparisons, showing a clear dominance of *Lactobacillus* and a reduction in anaerobic pathogens in the after group (Figure 4A). Receiver Operating Characteristic (ROC) analysis was employed to assess the predictive accuracy of these biomarkers. Several genera demonstrated high AUC values, including *Burkholderia* (AUC = 0.725), *Gardnerella* (AUC = 0.713), and *Segatella* (AUC = 0.703), indicating their potential as diagnostic indicators for therapeutic response (Figure 4B).

Spearman correlation analysis was performed to elucidate the ecological relationships among the key genera (Figure 4C). Notably, Lactobacillus exhibited strong negative correlations with a cluster of anaerobic bacteria, including Gardnerella, Sneathia, Prevotella, and Fannyhessea. These negative associations suggest that the restoration of Lactobacillus following Kushen Gel treatment may actively inhibit the growth of pathobionts through competitive exclusion or by modulating the local microenvironment, thereby facilitating a state conducive to HR-HPV clearance.

### 3.5 Clinical validation of Kushen Gel efficacy and microecological restoration

To bridge the gap between microbial findings and clinical outcomes, we conducted a prospective study. Initially, 50 patients with persistent HR-HPV infection were enrolled; however, 15 patients were subsequently excluded due to loss to follow-up or incomplete physiological data. Consequently, a Per-Protocol (PP) analysis was performed on the 35 patients who completed the full three-month intervention.

Following three menstrual cycles of Kushen Gel treatment, a therapeutic response was observed. The HR-HPV DNA positive rate plummeted from 100.0% at baseline to 17.1% at the follow-up visit. This represents an overall negative conversion rate of 82.9% (29/35), indicating that Kushen Gel significantly promotes the clearance of persistent HR-HPV (*p* < 0.001, Table 1).

**Table 1:** Clinical characteristics and therapeutic outcomes of the prospective cohort (*n* = 35).

Parameter	Baseline (pre-treatment)	Follow-up (post-treatment)	*p*-value
HR-HPV DNA (positive %)	100% (35/35)	17.1% (6/35)	<0.001
Vaginal pH	4.85 ± 0.42	4.12 ± 0.35	<0.001
Nugent score normalization^a^ (*n*, %)	6 (17.1%)	30 (85.7%)	<0.001

^a^HR-HPV DNA positive rate was determined by clinical PCR assay. Vaginal pH was measured using standardized pH strips. Nugent score normalization is defined as the transition from a dysbiotic state (Nugent score > 3) at baseline to a healthy vaginal flora (Nugent score 0–3) at the 3-month follow-up.

The clinical efficacy was accompanied by a significant remodeling of the vaginal niche. The mean vaginal pH decreased from 4.85 ± 0.42 to 4.12 ± 0.35 (*p* < 0.001), successfully restoring the healthy acidic buffer system. Categorical analysis of the Nugent scores further supported this restoration: the proportion of patients with a “Normal” vaginal flora (Nugent score 0–3) surged from 17.1% (6/35) to 85.7% (30/35) post-treatment (*p* < 0.001).

**Figure 5:**
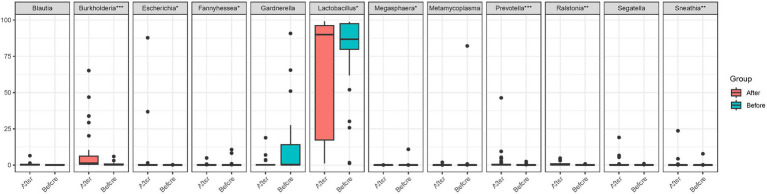
Paired longitudinal analysis of microbial changes in the prospective cohort following Kushen Gel treatment. *p < 0.05, **p < 0.01, ***p < 0.001.

Besides, paired 16S rRNA sequencing of these individuals post-treatment confirmed a dramatic ecological transition that closely mirrored the trends observed in the large-scale retrospective cohort (Figure 5). Specifically, the relative abundance of Lactobacillus significantly increased, while key pathobionts—including Gardnerella, Sneathia, and Prevotella—showed a consistent and marked decline. This synchronization of viral clearance and niche restoration within the same individuals provides robust evidence that Kushen Gel facilitates HR-HPV elimination by suppressing anaerobic pathogens and fostering a Lactobacillus-dominant environment, thereby strengthening the biological mucosal barrier.

### 3.6 Association between Kushen Gel treatment and a shift toward an anti-viral immune microenvironment

**Figure 6:**
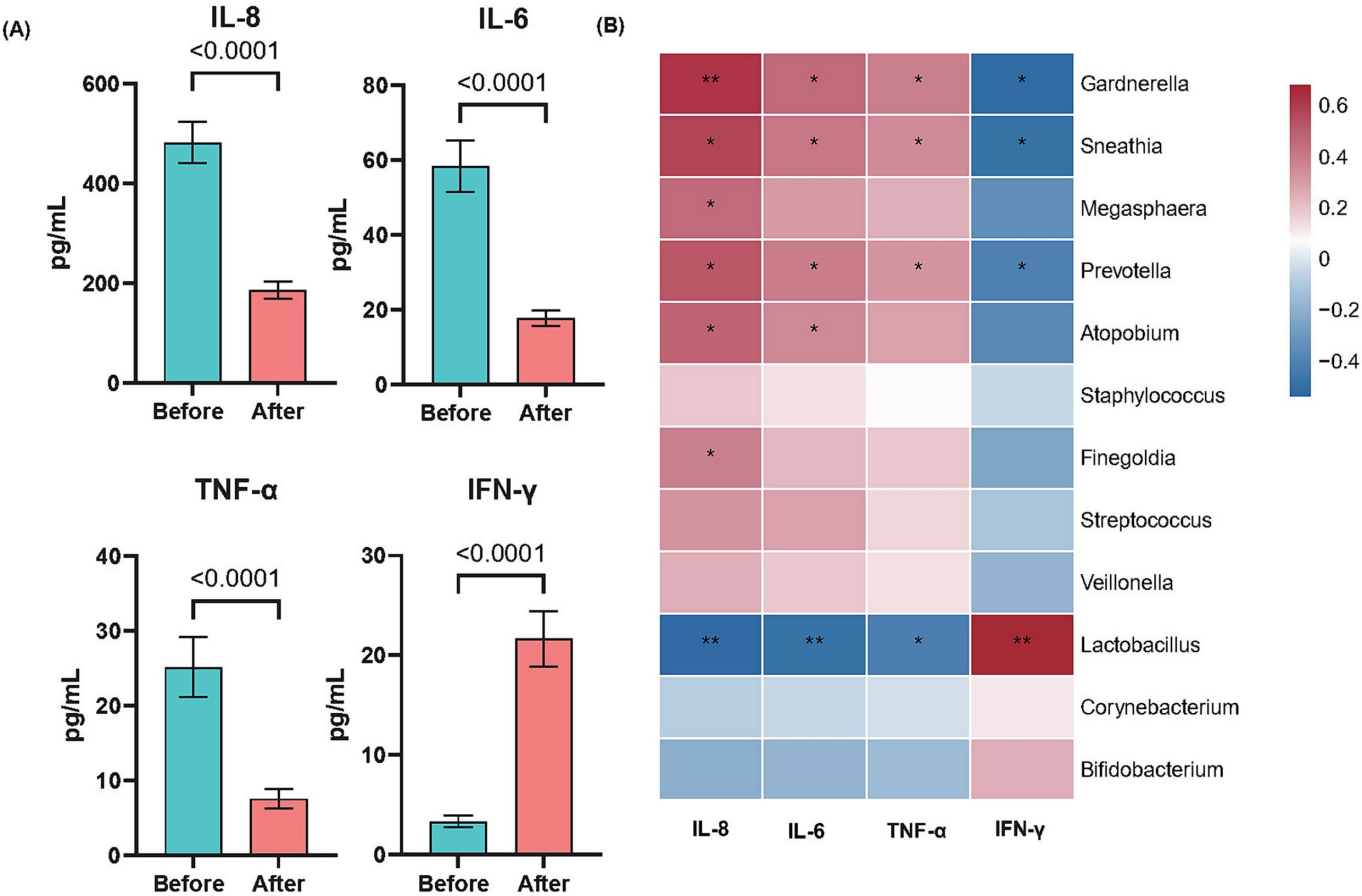
Kushen Gel orchestrates a transition from a pro-inflammatory to an anti-viral localized immune microenvironment. **(A)** Quantification of cervicovaginal cytokines before and after Kushen Gel treatment. **(B)** Spearman correlation heatmap elucidating the ecological relationships between dominant vaginal microbial genera and localized cytokine profiles. *p < 0.05, **p < 0.01, ***p < 0.001.

To bridge microbial restoration with clinical outcomes, we assessed cervicovaginal cytokine dynamics. Kushen Gel intervention triggered a profound shift in the vaginal immune microenvironment, transitioning from a pro-inflammatory state to a robust anti-viral profile. As shown in Figure 6A, concentrations of pro-inflammatory markers IL-8, IL-6, and TNF-*α* plummeted significantly post-treatment (*p* < 0.0001), while the anti-viral cytokine IFN-*γ* exhibited a robust increase (3.2 ± 1.1 to 18.6 ± 5.4 pg/mL, *p* < 0.0001), particularly in responders. Spearman correlation analysis (Figure 6B) confirmed that Lactobacillus abundance was positively associated with IFN-γ (r = 0.68), while being inversely correlated with IL-8 (r = −0.54) and other anaerobic pathobionts. These findings suggest that Kushen Gel facilitates HR-HPV clearance by suppressing anaerobic-driven inflammation and an enhanced IFN-γ-associated anti-viral microenvironment.

## 4 Discussion

The vaginal microenvironment is a complex ecosystem where the balance between commensal bacteria and the host immune system determines the natural history of HPV infection (22). In this study, we combined a large-scale retrospective 16S rRNA sequencing analysis with a prospective clinical cohort to evaluate the impact of Kushen Gel on the vaginal microbiota and HR-HPV clearance. Our study systematically investigates the mechanism by which Kushen Gel promotes high-risk HPV clearance, innovatively integrating microbial remodeling with localized immune shifts. Our results demonstrate that Kushen Gel intervention is associated with a significant reshaping of the vaginal microbial community, characterized by the enrichment of Lactobacillus and the suppression of anaerobic pathobionts, ultimately facilitating a physiological environment conducive to viral clearance.

The hallmark of a healthy vaginal environment is the dominance of Lactobacillus species, which maintain a low pH through the production of lactic acid and secrete antimicrobial compounds like hydrogen peroxide and bacteriocins (23, 24). Our retrospective analysis revealed a significant increase in the relative abundance of Lactobacillus following Kushen Gel treatment. This shift is critical, as Lactobacillus-derived lactic acid has been shown to inhibit the entry of HPV into cervical epithelial cells by maintaining mucosal integrity and modulating local immune responses (25, 26). The observed decrease in alpha diversity (ACE and Chao1 indices) post-treatment further supports the transition from a highly diverse, dysbiotic state to a stable, Lactobacillus-dominated state, a trend consistently associated with a lower risk of CIN progression (27, 28).

Persistent HR-HPV infection is frequently associated with high microbial diversity and the overgrowth of anaerobic bacteria such as *Gardnerella*, *Sneathia*, and *Fannyhessea* (29, 30). Our LEfSe and Random Forest models identified these genera as key biomarkers for the pre-treatment state. *Gardnerella* and *Sneathia* are known to produce enzymes (such as sialidases) and metabolites that degrade the protective cervical mucus layer, thereby facilitating viral access to the basal basement membrane (31, 32). Furthermore, *Sneathia* has been implicated in inducing pro-inflammatory cytokines that may promote cervical carcinogenesis (33).

Crucially, our study bridged the gap between microbial remodeling and biological efficacy by characterizing the localized immune microenvironment. The significant reduction in pro-inflammatory cytokines, including IL-8, IL-6, and TNF-*α*, following Kushen Gel treatment reflects a mitigation of the chronic inflammatory state typically driven by anaerobic dysbiosis. High levels of these markers have been shown to compromise mucosal integrity, our findings indicate that Kushen Gel-mediated suppression of pathobionts effectively “cools down” this inflammatory cascade, while the robust surge in IFN-*γ*—a master regulator of anti-viral immunity—was synchronized with HR-HPV clearance (34, 35). More importantly, the robust surge in IFN-*γ*—a master regulator of Th1-type anti-viral immunity—was synchronized with HR-HPV clearance. The significantly higher IFN-*γ* induction observed in Responders compared to Non-responders, coupled with its strong positive correlation with *Lactobacillus* abundance, provides compelling evidence that Kushen Gel does not merely act as an antimicrobial agent. While the robust elevation of IFN-γ suggests a potential enhancement of anti-viral defenses, it is important to note that our focused panel of four cytokines (IL-8, IL-6, TNF-*α*, IFN-γ) is insufficient to definitively characterize a complete Th1/Th2 paradigm shift. Rather, these findings indicate that Kushen Gel is associated with an ecological transition that fosters an IFN-γ-associated anti-viral immune niche—characterized by elevated IFN-γ and low inflammatory stress—which may collectively support the host’s ability to eliminate persistent viral particles.

The significant reduction of these pathobionts following Kushen Gel treatment aligns with the high HR-HPV negative conversion rate observed in our prospective cohort. Kushen Gel, containing matrine and oxymatrine, possesses potent antimicrobial properties that selectively inhibit anaerobic growth while sparing or supporting Lactobacillus colonization (16, 36). Our correlation analysis confirmed a strong negative relationship between Lactobacillus and these anaerobes, suggesting that Kushen Gel facilitates competitive exclusion, where Lactobacillus outcompetes pathobionts for nutrients and adhesion sites.

The prospective portion of our study provided crucial clinical validation. The significant reduction in vaginal pH and improved Nugent scores directly correlate with the taxonomical shifts observed via sequencing. A high vaginal pH (>4.5) is a known independent risk factor for HPV persistence, as it impairs the host’s innate immune defense (37, 38). By restoring a low-pH environment, Kushen Gel likely enhances the mucosal barrier’s function.

Our longitudinal analysis further reinforces this observation, as the transition from a high-diversity dysbiotic state to a Lactobacillus-dominant environment was synchronized with an 82.9% HR-HPV clearance rate. This finding is consistent with recent prospective cohorts suggesting that the restoration of Lactobacillus species, particularly *L. crispatus*, creates a protective “bio-shield” through the production of high concentrations of L-lactic acid and bacteriocins, which have been shown to directly inactivate viral particles and inhibit their entry into the cervical epithelium (25, 39).

Furthermore, the dramatic decrease in Nugent scores from intermediate or BV-associated levels to normal ranges in 85.7% of our participants underscores the potency of Kushen Gel in eradicating pathobionts. Unlike broad-spectrum antibiotics that may cause secondary dysbiosis, the matrine and oxymatrine components of Kushen Gel appear to exert selective antimicrobial pressure, specifically targeting anaerobic genera such as Gardnerella and Sneathia while sparing beneficial commensals (40, 41). This selective modulation is vital, as the reduction of anaerobic metabolites and sialidases prevents the degradation of cervical mucus, thereby maintaining the structural integrity of the mucosal barrier and facilitating the host’s innate immune recognition of HR-HPV (39, 42). The high degree of consistency between our retrospective findings and prospective validation reinforces that microbial niche remodeling represents a central pathway associated with Kushen Gel’s therapeutic profile.

Interestingly, our ROC analysis highlighted Burkholderia and Segatella as highly predictive of treatment response. While Lactobacillus is the primary beneficial taxon, the role of these emerging biomarkers in the vaginal niche warrants further investigation. Some studies suggest that certain non-pathogenic Proteobacteria may play a role in environmental buffering, though their specific contribution to HPV clearance remains to be fully elucidated (6, 43).

Several limitations of this study should be acknowledged. First, our prospective validation cohort specifically enrolled patients with documented persistent HR-HPV infection. While this underscores the efficacy of Kushen Gel in managing refractory viral persistence, these findings may not be directly extrapolated to patients with incident or transient infections, who might achieve clearance through natural immune responses without intervention. Future studies should include broader cohorts to validate these findings across different infection stages. Second, our findings demonstrate concurrent microbial and immune shifts, but they do not prove that microbiome alterations directly caused the immune changes. It remains plausible that immune restoration facilitated microbiome normalization, or that both processes were co-driven by the intervention. Third, although we describe an anti-viral immune shift, our analysis was limited to four cytokines (IL-8, IL-6, TNF-*α*, and IFN-*γ*). This limited panel is insufficient to characterize a complete Th1-skewed immune response, warranting broader immunological profiling in future research. Furthermore, differences in therapeutic responses across various HPV subtypes were not stratified. Different high-risk HPV genotypes, such as HPV16, HPV18, and others, may have distinct persistence patterns and immune evasion mechanisms, potentially affecting the efficacy of Kushen Gel. Future studies should incorporate subtype-specific analyses to evaluate whether microbial and immune remodeling differs across HPV subtypes, providing more precise insights into individualized therapy. Another limitation pertains to long-term efficacy. Our current follow-up was limited to three menstrual cycles, and it remains unclear whether the restored Lactobacillus-dominant state and the enhanced IFN-*γ*-associated anti-viral microenvironment are sustained over extended periods. Longitudinal monitoring beyond six to twelve months is warranted to assess the durability of HR-HPV clearance and potential recurrence, providing more comprehensive evidence for the clinical utility of Kushen Gel.

In conclusion, this study provides comprehensive evidence that Kushen Gel effectively remodels the vaginal microenvironment in patients with persistent HR-HPV infection. By leveraging a combined retrospective and prospective design, we demonstrated that Kushen Gel treatment triggers a profound ecological transition, characterized by the robust enrichment of beneficial Lactobacillus species, the concurrent suppression of anaerobic pathobionts, and the establishment of an IFN-γ- associated anti-viral immune profile. These findings provide high-quality evidence supporting the multi-target regulation of the “microbiota-immune” axis by traditional Chinese medicine and underscore the need for further investigation into HPV subtype-specific responses and long-term efficacy.

## Data Availability

The datasets presented in this study can be found in online repositories. The names of the repository/repositories and accession number(s) can be found in the article/supplementary material.
